# Augmented and mixed reality in liver surgery: a comprehensive narrative review of novel clinical implications on cohort studies

**DOI:** 10.1590/1806-9282.20250315

**Published:** 2025-07-07

**Authors:** Jan Roman, Ilker Sengul, Martin Němec, Demet Sengul, Marek Penhaker, Petr Strakoš, Petr Vávra, Ján Hrubovčák, Anton Pelikán

**Affiliations:** 1 University Hospital Ostrava, Department of General Surgery – Ostrava, The Czech Republic.; 2 University of Ostrava, Faculty of Medicine, Department of Surgical Studies – Ostrava, The Czech Republic.; 3 Giresun University, Faculty of Medicine, Division of Endocrine Surgery – Giresun, Turkey.; 4 Giresun University, Faculty of Medicine, Department of Surgery – Giresun, Turkey.; 5 VSB–Technical University of Ostrava, Faculty of Electrical Engineering and Computer Science – Ostrava, The Czech Republic.; 6 Giresun University, Faculty of Medicine, Department of Pathology – Giresun, Turkey.; 7 VSB–Technical University of Ostrava, Department of Cybernetics and Biomedical Engineering – Ostrava, The Czech Republic.; 8 VSB–Technical University of Ostrava, IT4Innovations National Supercomputing Center, – Ostrava, The Czech Republic.; 9 Tomas Bata University, Faculty of Humanities, Department of Health Care Sciences – Zlin, The Czech Republic.

## INTRODUCTION

Augmented reality (AR), *per se*, is a promising technology that facilitates surgical work in routine clinical practice. It superimposes computer-processed data (commonly a three-dimensional [3D] model based on preoperative imaging) onto authentic imagery using a computer device, usually an optical headset or a computer tablet. This adds new strata of perception without obstructing the view of the operative field. However, by definition, AR does not allow users to manipulate the displayed data. This is only possible in mixed reality (MR), enabling interactions between virtual and real elements in the field of view. This allows the operator to manipulate the presented computer-generated models, which can be further modified in real time synchronously with changes in the real scene^1,2^.

Due to high flexibility and broad possibilities, many surgical specialties have adopted these technologies quickly (i.e., neurosurgery and orthopedics). However, abdominal surgery, especially liver surgery, poses a significant challenge, as abdominal organs are generally variable in morphology and size and are subject to various intraoperative deformations, leading to difficulties accurately overlaying the virtual image onto real scenery. The lack of reliable anatomical landmarks on the liver surface and complicated intraparenchymal anatomy led to the development of various navigational systems. However, none (except for intraoperative ultrasonography) were widely accepted. Currently, advancements in mixed or AR are often seen as the most promising way to improve liver surgery's safety, duration, and comfort in everyday practice^1-3^.

## METHODS

A narrative review of available literature was performed using the search terms "augmented reality," "mixed reality," and "liver" in the PubMed/MEDLINE database. The initial search was conducted during April 2024 and yielded 173 published records. Further, 11 references were gathered using cross-referencing. Duplicates, reviews, conference abstracts, commentaries, video reports, and papers describing only virtual reality or interventional procedures were excluded, as well as technological papers without clinical relevance. Only the English-language literature was included (except for two Chinese consensus publications). After manually examining the remaining records and abstracts, 63 relevant studies were included in the review.

## RESULTS

**Figure 1:**
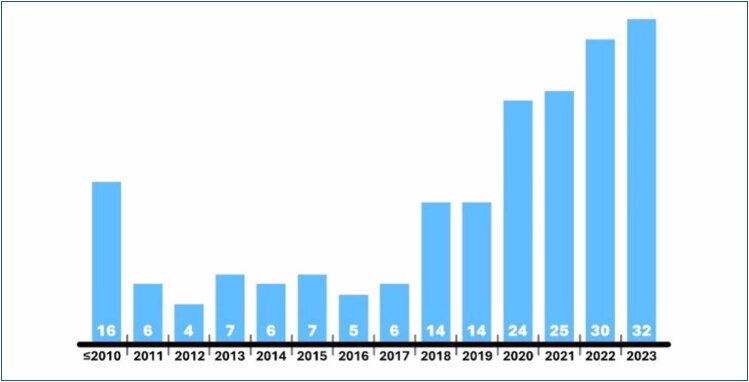
Number of publications in individual years detected by the initial search.

It has been apparent that AR technology in liver surgery is undergoing rapid development. The total number of publications found by the initial search gradually rises yearly, as shown in Figure 1, from initial rare reports in 2005 to tens of papers published in 2023. The AR/MR applications are being increasingly accepted as a newly emerging and inseparable part of clinical hepatic surgery, especially in Asian countries with a high incidence of hepatic cancer. This has resulted in the first expert consensus for future research and clinical applications^1^.

### Augmented reality/mixed reality implementation

While AR can be utilized in both open and laparoscopic/robotic surgeries, the implementation of AR into laparoscopic liver resection (LLR) is considered more advantageous than open liver resection (OLR) implementation. Currently, the high field of view and the high resolution of laparoscopic cameras provide an excellent actual image for virtual overlays. Furthermore, the view is less obstructed by surrounding tissues and the abdominal wall. Another benefit is the absence of an external screen to display the overlay, as it can be projected directly onto the laparoscopic monitor. On the other hand, features of MR cannot be easily implemented as 3D manipulation with the virtual scene is more complicated. It is important to note that AR/MR can also be helpful during open surgery, as reported by many authors, even during complex procedures, where AR was successfully used during an ALPPS procedure in a patient with numerous liver metastases.^2-5^

Even though most AR surgery research focuses on perioperative navigation, Saito et al.^3^ reported that most surgeons value AR during surgery for last-minute exploration of the 3D model instead of proper navigation during surgery. It was also reported that AR can decrease the time necessary for determining the location of the lesion inside the respective segment four or the position of trocars when paired with projection to the patient's body surface or with a conventional AR display. By displaying a sonographic image onto a laparoscopic view with proper alignment, AR can be used to guide the needle during perioperative ablation^6^. Ntourakis et al.^7^ presented a small case series where AR was used to localize sites of missing liver metastases after complete pathologic remission.

### Technological notes

During the initialization of AR/MR systems, calibration and/or registration are required to align virtual and real images precisely, including the calibration of the laparoscopic camera. On the whole, tissue deformation is the most challenging aspect of proper image visualization and alignment. Using physical models to compensate for deformation is complicated as many forces are acting on the liver, such as direct manipulation, pneumoperitoneum pressure, and movement of the diaphragm, heart, and bowels. Currently, most authors use repeated perioperative registration of images to compensate for these limitations.

For registration purposes, many approaches can be found in the literature. As the liver surface is primarily homogeneous, specific landmarks are usually used, and the edges of the liver are employed most frequently. Pelanis et al.^8^ proposed using fiducials injected into the liver parenchyma with fluoroscopic registration updates. Falkenberg et al.^9^ used radiopaque fiducials implanted under sonographic guidance for the same purpose. Golse et al.^10^ devised a non-rigid registration system using a physics-based elastic model to account for organ deformation in real time. While this system achieved sufficient internal precision of<1 cm, the authors describe subpar performance with current hardware (5–7 frames per second). The biomechanical properties of heterogeneous liver tissues were also used in the work by Haouchine et al.^11^ with satisfactory results. A combination of manual rigid registration and an automatic biomechanical model was proposed by Espinel et al.^12^ in a novel hybrid registration system.

In order to experimentally evaluate the achieved precision of the registration and visualization, many methods were developed: the re-projection error (RPE)^13^, various types of registration errors (REs)^14-17^, visualization errors (VEs)^18^, and a pointing error (PE)^19^. Commonly, the definition of these methods is not uniform, and therefore, direct comparisons between individual papers are not possible. Furthermore, more advanced methods for image alignment using deep learning methods have been introduced in recent years, with improved precision. The best achievable precision is made possible using ex-vivo phantoms and can reach submillimeter values^20^.

Using AR and MR can cause unwanted side effects in specific individuals. These include headache, nausea, dizziness, and eye fatigue. These are commonly attributed to the "fluency" of the system, mainly referring to achieved frames per second and visualization delay. AR and MR visualizations often require powerful hardware to achieve sufficient performance. A lack of proper depth perception further complicates the matter. Even though headsets can provide operators with 3D images, the combination of a real scene with translucent AR image results cannot show the depth properly. Methods to deal with this problem are being developed, but no single method alone could provide a good depth of perception. AR image puts a significant cognitive load on the surgeon as more information must be processed^21,22^. A negative effect called "inattentional blindness" was examined by Dixon et al., describing more difficulties in identifying significant unexpected findings clearly during AR-assisted surgery^23^. To address the possibility of mental overload, context-aware AR systems were developed^24^. The cognitive hurdles are present, especially when using AR headsets instead of a conventional screen, and according to Condino et al.^25^, it is imperative to focus on improving the AR technology to alleviate these problems.

While displaying an AR/MR image onto an actual image, there is a complete overlay of the 3D image. An opaque 3D model was used by Conrad et al.^26^; however, this opacity proved not ideal for instrument targeting. In most studies, semitransparent models have been used to solve this issue. Furthermore, surgeons prefer to see the instruments as clearly as possible, which is not possible using a regular 3D model placement. To address this problem, Hofman et al.^27^ used artificial intelligence (AI) to remove the overlaying parts of the 3D image. The AI system was trained on manually annotated data, also demonstrated during liver surgery. AI was also implemented by Kasai et al.^28^, where it was used to outline liver contour in the recorded image and therefore improve overall accuracy with lower RE (14.5 vs. 31.2 mm).

### Clinical outcomes

Currently, AR systems are available that allow for routine AR implementation during surgery. As a result, more authors report the results of AR-assisted surgical procedures. However, most of these systems are proprietary, developed, and utilized by the same team and institution. Of note is that an increased number of authors present case reports of using AR during OLR or LLR. All authors agree that this technology allows for more precise and safe surgery, particularly when combined with indocyanine green (ICG) administration.

The results of single-cohort studies utilizing AR assistance during procedures are presented in Table 1. It is evident from the given data that the short-term outcomes of AR-assisted liver resections are on par or better compared to AR-unassisted procedures. Furthermore, AR assistance may provide surgeons with greater accuracy, as Tao et al.^30^ demonstrated. This study had a higher agreement between predicted and actual liver resection volume (absolute error: 26.6 vs. 51.8 mL, p<0.0001). This attenuation of the amount of liver parenchyma removed can benefit patients. AR overlay can be helpful during segment identification when ICG administration does not correctly reveal segment boundaries, as presented by Deng et al.^32^

**Table 1:** Clinical outcomes of single-cohort studies.

Authors (year)	N	Procedure	ICG	Blood loss median [mL]	Duration of surgery [min]	Duration of hospital stay [days]	Complication rate [%]
Laparoscopic cohorts							
Deng et al.^29^	16	Left hemihepatectomy	Yes	116.3±64.5	380.3±92.2	8.2±2.7	31.3
Wang et al.^33^	11	Right hemihepatectomy+caudate lobectomy	No	209.1±56.1	454.5±25.0	10.5±1.2	45.5
Naito et al.^34^	6	Minor resections	No	178.5 (100–1,000)	433 (337–597)	12 (8–20)	33.3
Zhu et al.^35^	5	Narrow right hemihepatectomy	No	–	300 (270–360)	8 (7–9)	–
Prevost et al.^14^	9	Non-anatomical resections+right hemihepatectomy	No	325 (20–1,200)	128	–	–
Bertrand et al.^36^	17	Various	No	260 (I200–500)	260 (210–360)	6 (5–8)	10 (only severe)

ICG: indocyanine green.

Several authors have verified that AR improves perioperative outcomes compared to surgery without AR assistance, especially when combined with other navigational methods. The results of such studies are shown in Table 2. Most authors reported a statistically significant reduction in blood loss and transfusion administration. Wang et al.^37^ showed a lower rate of remnant liver ischemia (13.3 vs. 30.2%) and disease-free survival rate (70.01 vs. 52.46% at 3 years of follow-up) after laparoscopic segmentectomy for hepatocellular carcinoma. There was no significant difference in recurrence rate between AR-assisted and non-AR-assisted resection after 16 months of follow-up after primary liver cancer, as reported by Zhang et al.^38^. In addition, Zhu et al.^39^ emphasized that employment of MR in liver resection significantly lowers the time of portal vein obstruction due to the Pringle maneuver (17.71±4.16 min in MR-assisted resection vs. 21.58±5.24 min without MR, p=0.019). Finally, Wu et al.^40^ successfully used AR during hepatectomy for hepatolithiasis, resulting in significantly lower concentrations of alanine transaminase and aminotransferase postoperatively, as well as a decrease in the rate of residual stones (19.4 vs. 41.3%). Last but not the least, hepatocellular health is significantly crucial for human beings^41,42^.

**Table 2:** Clinical outcomes of comparative studies (values reported as non-augmented reality-group/augmented reality-group).

Authors (year)	n	Procedure	ICG	Blood loss median [mL]	Duration of surgery [min, rounded]	Transfusion rate [%]	Duration of hospital stay [days, rounded]	Complication rate [%]
Laparoscopic cohorts								
Wang et al.^37^	45/53	Anatomical segmentectomy	Yes	100/200 (p=0.005)	290/240 (p=0.086)	6.7/13.2 (p=0.335)	7/7 (p=0.788)	31.1/49.1 (p=0.072)
Toman et al. ^41^	42/36	Central hepatectomy	Yes	275/300 (p=0.013)	313/323 (p=0.660)	14.3/64.7 (p=0.010)	8/9 (p=0.005)	35.7/61.8 (p=0.024)
Tao et al.^30^	16/15	S8 (sub)segmentectomy	Yes	125/300 (p=0.003)	383/359 (p=0.640)	18.8/33.3 (p=0.433)	9/9 (p=0.258)	12.5/26.7 (p=0.394)
Wu et al.^40^	31/46	Various	Yes	113/207 (p<0.001)	367/272 (p<0.001)	12.9/17.4 (p=0.753)	9/8 (p=0.143)	25.8/32.6 (p=0.522)
Zhang et al.^38^	44/41	Various	No	200/300 (p=0.002)	300/300 (p=0.061)	10/42 (p<0.001)	8/10 (p=0.003)	41/46 (p=0.614)
Mixed cohorts								
Huber et al.^31^	8/12	Minor resections	No	–	190/185 (p=0.970)	–	9/7 (p=0.368)	12.5/8.3 (p=0.696)
Open cohorts								
Zhu et al.^39^	37/58	Various	Both	329/398 (p=0.010)	203/230 (p=0.003)	–	12/14 (p=0.049)	18.9/39.7 (p=0.034)

ICG: indocyanine green.

## CONCLUSION

The current state of AR and MR shows their potential to enhance everyday surgery for healthcare providers. Current clinical data from cohort studies suggest that with AR and MR surgery could be safer, faster, and more comfortable. However, before AR and MR can be implemented into everyday surgical practice, several technological challenges must be addressed to improve ergonomics, image perception, and the mental load it places on surgeons.

## Data Availability

The datasets generated and/or analyzed during the current study are available from the corresponding author upon reasonable request.

## References

[1] Hepatic Surgery Group, Surgery Branch of Chinese Medical Association, Digital Medical Branch of Chinese Medical Association, Digital Intelligent Surgery Committee of Chinese Research Hospital Association, Liver Cancer Committee of Chinese Medical Doctor Association (2023). [Chinese expert consensus on laparoscopic hepatic segmentectomy and subsegmentectomy navigated by augmented and mixed reality technology combined with indocyanine green fluorescence(2023)]. Chin Med Assoc Publ House.

[2] Fidan D, Mero G, Mazilescu LI, Heuer T, Kaiser GM (2023;). Mixed reality combined with ALPPS for colorectal liver metastases, a case report. Int J Surg Case Rep.

[3] Saito Y, Sugimoto M, Imura S, Morine Y, Ikemoto T, Iwahashi S (2020). Intraoperative 3D hologram support with mixed reality techniques in liver surgery. Ann Surg.

[4] Pelanis E, Kumar RP, Aghayan DL, Palomar R, Fretland ÅA, Brun H (2020). Use of mixed reality for improved spatial understanding of liver anatomy. Minim Invasive Ther Allied Technol.

[5] Volonté F, Pugin F, Bucher P, Sugimoto M, Ratib O, Morel P (2011). Augmented reality and image overlay navigation with OsiriX in laparoscopic and robotic surgery: not only a matter of fashion. J Hepatobiliary Pancreat Sci.

[6] Liu X, Plishker W, Kane TD, Geller DA, Lau LW, Tashiro J (2020). Preclinical evaluation of ultrasound-augmented needle navigation for laparoscopic liver ablation. Int J Comput Assist Radiol Surg.

[7] Ntourakis D, Memeo R, Soler L, Marescaux J, Mutter D, Pessaux P (2016). Augmented reality guidance for the resection of missing colorectal liver metastases: an initial experience. World J Surg.

[8] Pelanis E, Teatini A, Eigl B, Regensburger A, Alzaga A, Kumar RP (2021;). Evaluation of a novel navigation platform for laparoscopic liver surgery with organ deformation compensation using injected fiducials. Med Image Anal.

[9] Falkenberg M, Rizell M, Sternby Eilard M, Regensburger A, Razazzian R, Kvarnström N (2021). Radiopaque fiducials guiding laparoscopic resection of liver tumors. Surg Laparosc Endosc Percutan Tech.

[10] Golse N, Petit A, Lewin M, Vibert E, Cotin S (2021). Augmented reality during open liver surgery using a markerless non-rigid registration system. J Gastrointest Surg.

[11] Haouchine N, Cotin S, Peterlik I, Dequidt J, Lopez MS, Kerrien E (2015). Impact of soft tissue heterogeneity on augmented reality for liver surgery. IEEE Trans Vis Comput Graph.

[12] Espinel Y, Özgür E, Calvet L, Le Roy B, Buc E, Bartoli A (2020). Combining visual cues with interactions for 3D-2D registration in liver laparoscopy. Ann Biomed Eng.

[13] Thompson S, Schneider C, Bosi M, Gurusamy K, Ourselin S, Davidson B (2018). In vivo estimation of target registration errors during augmented reality laparoscopic surgery. Int J Comput Assist Radiol Surg.

[14] Prevost GA, Eigl B, Paolucci I, Rudolph T, Peterhans M, Weber S (2020). Efficiency, accuracy and clinical applicability of a new image-guided surgery system in 3D laparoscopic liver surgery. J Gastrointest Surg.

[15] Fitzpatrick JM, West JB (2001). The distribution of target registration error in rigid-body point-based registration. IEEE Trans Med Imaging.

[16] Mountney P, Fallert J, Nicolau S, Soler L, Mewes PW (2014). An augmented reality framework for soft tissue surgery. Med Image Comput Comput Assist Interv.

[17] Yasuda J, Okamoto T, Onda S, Futagawa Y, Yanaga K, Suzuki N (2018). Novel navigation system by augmented reality technology using a tablet PC for hepatobiliary and pancreatic surgery. Int J Med Robot.

[18] Wild E, Teber D, Schmid D, Simpfendörfer T, Müller M, Baranski AC (2016). Robust augmented reality guidance with fluorescent markers in laparoscopic surgery. Int J Comput Assist Radiol Surg.

[19] Ribeiro M, Espinel Y, Rabbani N, Pereira B, Bartoli A, Buc E (2024). Augmented reality guided laparoscopic liver resection: a phantom study with intraparenchymal tumors. J Surg Res.

[20] Shao L, Yang S, Fu T, Lin Y, Geng H, Ai D (2022;). Augmented reality calibration using feature triangulation iteration-based registration for surgical navigation. Comput Biol Med.

[21] Paolis LT, Luca V (2019). Augmented visualization with depth perception cues to improve the surgeon's performance in minimally invasive surgery. Med Biol Eng Comput.

[22] Hansen C, Wieferich J, Ritter F, Rieder C, Peitgen HO (2010). Illustrative visualization of 3D planning models for augmented reality in liver surgery. Int J Comput Assist Radiol Surg.

[23] Dixon BJ, Daly MJ, Chan H, Vescan AD, Witterick IJ, Irish JC (2013). Surgeons blinded by enhanced navigation: the effect of augmented reality on attention. Surg Endosc.

[24] Katić D, Wekerle AL, Görtler J, Spengler P, Bodenstedt S, Röhl S (2013). Context-aware augmented reality in laparoscopic surgery. Comput Med Imaging Graph.

[25] Condino S, Carbone M, Piazza R, Ferrari M, Ferrari V (2020). Perceptual limits of optical see-through visors for augmented reality guidance of manual tasks. IEEE Trans Biomed Eng.

[26] Conrad C, Fusaglia M, Peterhans M, Lu H, Weber S, Gayet B (2016). Augmented reality navigation surgery facilitates laparoscopic rescue of failed portal vein embolization. J Am Coll Surg.

[27] Hofman J, Backer P, Manghi I, Simoens J, Groote R, Bossche H (2023). First-in-human real-time AI-assisted instrument deocclusion during augmented reality robotic surgery. Healthc Technol Lett.

[28] Kasai M, Uchiyama H, Aihara T, Ikuta S, Yamanaka N (2023). Laparoscopic projection mapping of the liver portal segment, based on augmented reality combined with artificial intelligence, for laparoscopic anatomical liver resection. Cureus.

[29] Deng H, Zeng X, Xiang N (2023). Augmented reality navigation system and indocyanine green fluorescence imaging make laparoscopic right anterior sectionectomy more precisely and safely. J Gastrointest Surg.

[30] Tao H, Wang Z, Zeng X, Hu H, Li J, Lin J (2023). Augmented reality navigation plus indocyanine green fluorescence imaging can accurately guide laparoscopic anatomical segment 8 resection. Ann Surg Oncol.

[31] Huber T, Tripke V, Baumgart J, Bartsch F, Schulze A, Weber S (2023). Computer-assisted intraoperative 3D-navigation for liver surgery: a prospective randomized-controlled pilot study. Ann Transl Med.

[32] Deng H, Zeng X, Hu H, Zeng N, Huang D, Wu C (2024). Laparoscopic left hemihepatectomy using augmented reality navigation plus ICG fluorescence imaging for hepatolithiasis: a retrospective single-arm cohort study (with video). Surg Endosc.

[33] Wang Z, Tao H, Wang J, Zhu Y, Lin J, Fang C (2023). Laparoscopic right hemi-hepatectomy plus total caudate lobectomy for perihilar cholangiocarcinoma via anterior approach with augmented reality navigation: a feasibility study. Surg Endosc.

[34] Naito S, Kajiwara M, Nakashima R, Sasaki T, Hasegawa S (2023). Application of extended reality (virtual reality and mixed reality) technology in laparoscopic liver resections. Cureus.

[35] Zhu W, Zeng XJ, Xiang N, Zeng N, Liu ZH, Fang XQ (2022). [Application of augmented reality and mixed reality navigation technology in laparoscopic limited right hepatectomy]. Zhonghua Wai Ke Za Zhi.

[36] Bertrand LR, Abdallah M, Espinel Y, Calvet L, Pereira B, Ozgur E (2020). A case series study of augmented reality in laparoscopic liver resection with a deformable preoperative model. Surg Endosc.

[37] Wang D, Hu H, Zhang Y, Wu X, Zeng X, Yang J (2024). Efficacy of augmented reality combined with indocyanine green fluorescence imaging guided laparoscopic segmentectomy for hepatocellular carcinoma. J Am Coll Surg.

[38] Zhang W, Zhu W, Yang J, Xiang N, Zeng N, Hu H (2021;). Augmented reality navigation for stereoscopic laparoscopic anatomical hepatectomy of primary liver cancer: preliminary experience. Front Oncol.

[39] Zhu LY, Hou JC, Yang L, Liu ZR, Tong W, Bai Y (2022). Application value of mixed reality in hepatectomy for hepatocellular carcinoma. World J Gastrointest Surg.

[40] Wu X, Zeng N, Hu H, Pan M, Jia F, Wen S (2022). Preliminary exploration on the efficacy of augmented reality-guided hepatectomy for hepatolithiasis. J Am Coll Surg.

[41] Toman D, Sengul I, Pelikán A, Sengul D, Vavra P, Ihnát P (2022). Hepatocellular carcinoma versus nonalcoholic fatty liver disease: metabolic, environmental, and genetic association? *De facto*?. Rev Assoc Med Bras (1992).

[42] Toman D, Sengul I, Pelikán A, Sengul D, Vavra P, Ihnat P (2022). A narrative review on nonalcoholic fatty liver disease and nonalcoholic steatohepatitis versus hepatocellular carcinoma: do you mind?. Rev Assoc Med Bras (1992).

